# Efficacy and Safety of Finerenone in Chronic Kidney Disease: A Systematic Review and Meta-Analysis of Randomized Clinical Trials

**DOI:** 10.3389/fphar.2022.819327

**Published:** 2022-02-07

**Authors:** Ming-Zhu Zhang, Wujisiguleng Bao, Qi-Yan Zheng, Ya-Hui Wang, Lu-Ying Sun

**Affiliations:** ^1^ Dongzhimen Hospital Affiliated to Beijing University of Chinese Medicine, Beijing, China; ^2^ Renal Research Institution of Beijing University of Chinese Medicine, Beijing, China; ^3^ Fangshan Hospital Affiliated to Beijing University of Chinese Medicine, Beijing, China

**Keywords:** finerenone, chronic kidney disease, systematic review, meta-analysis, randomized clinical trials

## Abstract

**Background:** Chronic kidney disease (CKD) is a global public health issue. In recent years, the effectiveness of finerenone for treatment of CKD has been the subject of considerable debate. The main objective of the current meta-analysis was to validate the clinical efficacy and safety of finerenone in patients with CKD.

**Methods:** Seven databases were searched for randomized controlled trials (RCTs) comparing finerenone with placebo in patients with CKD. Data from eligible studies were extracted, and the Cochrane risk of bias tool utilized for evaluating the methodological quality of RCTs. The effect size was estimated using the risk ratio (RR) and mean difference (MD) with 95% confidence interval (CI).

**Results:** Five trials (n = 13,078) were included. Compared to placebo groups, the urinary albumin-to-creatinine ratio (UACR) mean from the baseline was significantly lower [MD −0.30 (95% CI −0.32, −0.28), *p* < 0.00001], while a decrease in the estimated glomerular filtration rate (eGFR) from baseline was significantly higher [MD −2.44 (95% CI −2.82, −2.05), *p* < 0.00001] for the finerenone groups. Furthermore, the proportion of patients with decreased eGFR (≥40%) post-baseline was significantly lower [RR 0.85 (95% CI 0.78, 0.93), *p* = 0.0002], along with end-stage kidney disease (ESKD) [RR 0.80 (95% CI 0.65, 0.99), *p* = 0.04] and cardiovascular events (CVs) [RR 0.88 (95% CI 0.80, 0.95), *p* < 0.003] in the finerenone groups. In terms of safety, the increase in the serum potassium concentration and incidence of hyperkalemia was significantly higher for the finerenone groups [MD 0.17 (95% CI 0.10, 0.24), *p* < 0.00001; RR 2.03 (95% CI 1.83, 2.26), *p* < 0.00001, respectively], but the incidence of adverse events (AEs) was similar to placebo [RR 1.00 (95% CI 0.98–1.01), *p* = 0.67]. In all cases, the results were rated as providing moderate-quality or high-quality evidence.

**Conclusion:** Data from our meta-analysis suggest that finerenone confers significant renal and cardiovascular benefits in patients with CKD. While higher risk of hyperkalemia was observed with finerenone than placebo, differences in AEs were not significant. Finerenone may therefore present a novel promising therapeutic agent for patients with CKD.

**Systematic Review Registration**: [https://inplasy.com/inplasy-2021-9-0020/], identifier [INPLASY202190020].

## Introduction

Chronic kidney disease (CKD) is a major global health issue posing a heavy burden on society, with a reported estimated prevalence of 9.1% (GBD Chronic Kidney Disease Collaboration, 2020). Multiple factors are involved in the pathophysiological development of CKD, including aldosterone, a steroid hormone and downstream target of the renin angiotensin system (RAS) (Barrera-Chimal et al., 2019; Ingelfinger and Rosen, 2020). The adrenal steroid hormone binds the mineralocorticoid receptor (MR), promoting sodium retention and potassium loss and thereby controlling the electrolyte status. Considerable evidence supports a pathophysiological role of aldosterone via MR overactivation in CKD and cardiorenal diseases through induction of inflammation and fibrosis that leads to progressive kidney and cardiovascular dysfunction (Bakris et al., 2021; Epstein, 2021). In addition, cardiovascular disease (CVD) is the leading cause of death and morbidity in people with chronic kidney disease (Epstein, 2015). Strategies to protect the kidneys of patients with CKD may mitigate their risk of cardiovascular events (Filippatos et al., 2021).

Currently, the steroidal mineralocorticoid receptor antagonists (MRAs) including the first-generation spironolactone and the second-generation eplerenone are strongly recommended for patients with CKD and chronic heart failure (CHF) (Ponikowski et al., 2016; Pitt et al., 2017; Maddox et al., 2021). However, these agents remain underutilized, largely due to safety concerns regarding the potential development of hyperkalemia, worsening renal function, gynecomastia and impotence (in men), and menstrual disturbances (in women) (Bakris et al., 2021). More recently, third-generation MRA using non-steroidal molecules have been developed that more selectively enhance benefits and minimize risks owing to altered receptor affinity and tissue tropism (Bramlage et al., 2016). Finerenone (BAY 94-8662) is a novel potent, non-steroidal, selective MRA developed for the treatment of CKD (Georgianos and Agarwal, 2021). Compared with the currently available steroid-based MRAs, finerenone has greater selectivity for the MR over other steroid hormone receptors than spironolactone and improved affinity for MR in relation to eplerenone while maintaining very low affinity for androgen, glucocorticoid, and progesterone receptors, with more potent anti-inflammatory and antifibrotic efficacy in preclinical models (Bramlage et al., 2016; Grune et al., 2018). In addition, comparative preclinical studies have revealed favorable properties of cardiorenal end-organ protection (Kolkhof et al., 2014; Grune et al., 2018). Thus, finerenone may effectively address the unmet medical need for kidney and cardiovascular protection.

In recent years, several clinical researchers have focused on the efficacy and safety of finerenone. The FDA has approved finerenone to reduce the risk of kidney function decline, kidney failure, cardiovascular death, non-fatal heart attacks, and hospitalization for heart failure in adults with chronic kidney disease associated with type 2 diabetes (FDA, 2021). The current meta-analysis was performed to comprehensively evaluate the efficacy and safety of finerenone for CKD based on documented phase 2 and 3 randomized controlled trials (RCTs), with a view to providing substantial evidence for supporting its clinical application in a wide range of patients.

## Materials and Methods

This review complies with the PRISMA 2020 statement: an updated guidance and exemplars for reporting systematic reviews (Page et al., 2021a; b) (Supplementary Table S1).

### Search Strategy

We comprehensively searched PubMed, Embase, Cochrane Library, SinoMed, China National Knowledge Infrastructure (CNKI), WanFang, and Chongqing VIP Information databases from inception until September 2021 for RCTs investigating the utility of finerenone for CKD in adults. Additional studies were searched in the reference lists of all identified publications, including relevant meta-analyses and systematic reviews (Supplementary Table S2).

### Inclusion Criteria

The inclusion criteria were as follows: (I) study design: RCT, (II) comparison: evaluating the efficacy and safety of finerenone with that of a placebo, (III) population: patients with CKD, and (IV) outcome: assessment of at least one of the following outcomes: 1) primary outcomes: UACR from the baseline, change in eGFR; secondary outcomes: proportion of patients with ≥40% decrease in eGFR at any time post-baseline, proportion of patients with ESKD, CVs, changes in serum potassium concentration, proportion of patients with hyperkalemia, and 2) AEs.

### Data Extraction

Two reviewers (M-ZZ and WB) extracted data from the original trials independently. Data extracted included study characteristics (first author, publication year, location, sample size, intervention and control, period of treatment and duration of follow-up, and phase of clinical trial), characteristics of patients (inclusion criteria, mean age, proportion of men, baseline eGFR, and baseline UACR), reported outcomes (UACR, eGFR, ESKD, CV events, serum potassium concentration, hyperkalemia, and AEs), and information on methodology.

### Quality Assessment

The risk of bias of RCTs was assessed using the Cochrane Collaboration’s tool (Higgins et al., 2021). Two investigators (WB and M-ZZ) independently completed the assessments. If necessary, any disagreement was resolved by consensus with a third author (Y-HW). In addition, the Grading of Recommendations Assessment, Development, and Evaluation (GRADE) framework was employed to evaluate the quality of evidence contributing to each estimate. GRADE can be effectively applied to characterize the quality of a body of evidence on the basis of study limitations, imprecision, inconsistency, indirectness, and publication bias for all outcomes (Higgins et al., 2021).

### Statistical Analysis

Data entry and analysis were conducted using Excel 2019 (Microsoft, Redmond, WA, United States) and ReviewManager (RevMan) 5.4.1. The mean difference (MD) with 95% confidence interval (CI) of the outcomes and the risk ratio (RR) were calculated as the effect measure. The I^2^ statistic for heterogeneity was calculated as a measure of the proportion of the overall variation attributable to between-study heterogeneity. A fixed-effects model was chosen if I^2^<50%; otherwise, the random-effects model was used. A subgroup or sensitivity analysis was conducted to explore the underlying causes of heterogeneity in treatment outcomes. Publication bias was examined by visual inspection of the funnel plots. In cases where only a few studies are included in the analysis, the power of the tests is too low. Accordingly, publication bias was only examined if > 10 comparative studies were included for analysis (Higgins et al., 2021). Upon the inclusion of several subgroups for analysis (finerenone dose ≥10 mg/d), outcomes were pooled. In cases where standard deviations of UACR, eGFR, or serum potassium concentration could not be directly obtained from the trials, the mean and deviation were estimated from the confidence interval (Higgins et al., 2021).

## Results

### Study Characteristics

**FIGURE 1:**
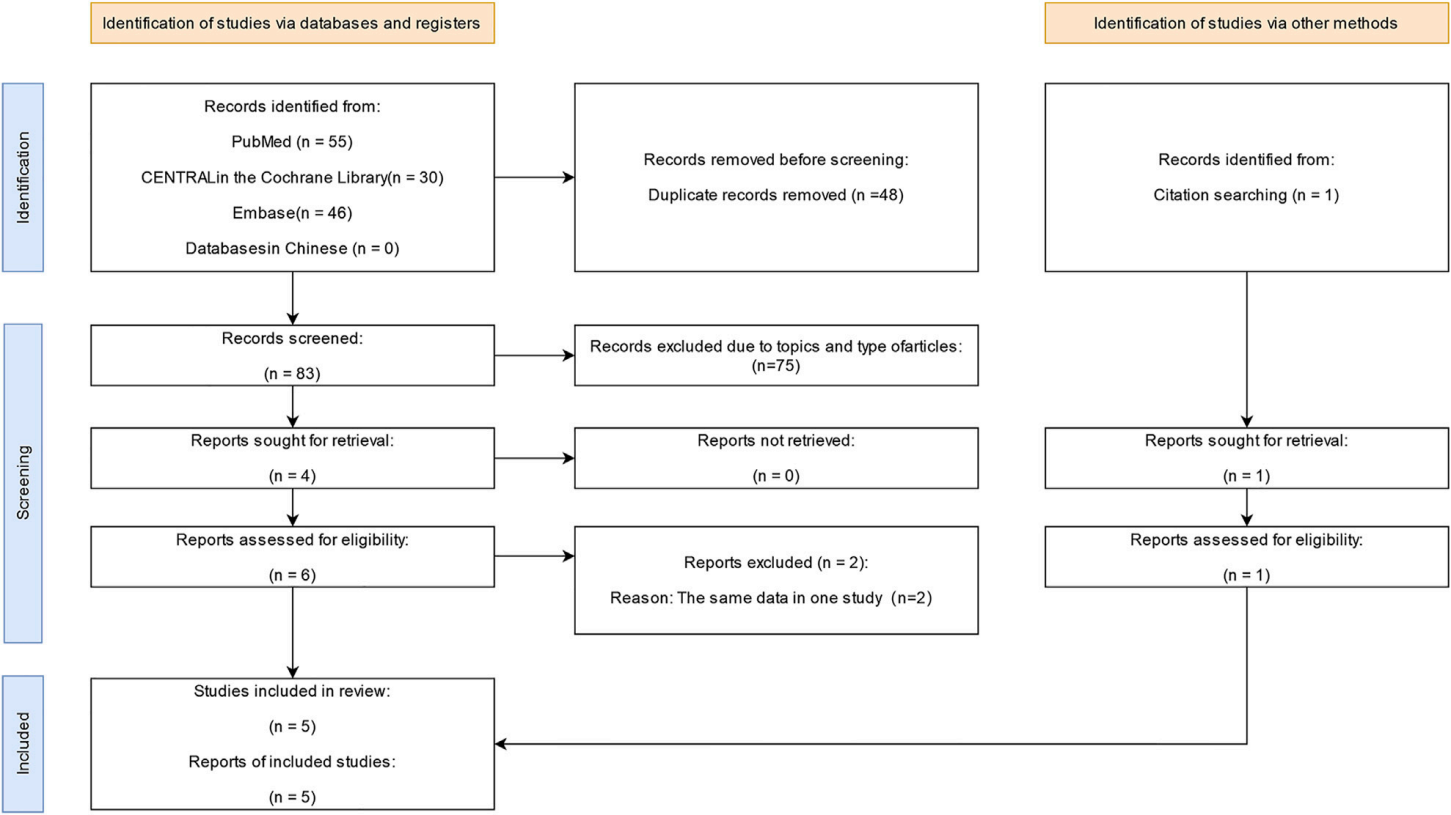
Flow diagram of the screening process.

Following our search, 132 relevant articles were identified, among which 48 were duplicates. Subsequently, 84 titles and abstracts were screened, with the full-text screening of seven articles. Finally, five eligible studies (Pitt et al., 2013; Bakris et al., 2015; Katayama et al., 2017; Bakris et al., 2020; Pitt et al., 2021) (14,335 participants) were included in our analysis of the efficacy and safety of finerenone for CKD. Figure 1 depicts the screening process, and Supplementary Table S3 presents the main characteristics of the included trials. One trial (Pitt et al., 2013) enrolled patients with CKD and heart failure with reduced ejection fraction (HFrEF), one (Bakris et al., 2015) enrolled patients with diabetic nephropathy (DN), one (Katayama et al., 2017) enrolled patient with DN and type 2 diabetes mellitus (T2DM), and the other two (Bakris et al., 2020; Pitt et al., 2021) enrolled patients with CKD and T2DM. All trials reported the clinical benefit of finerenone and adverse events in patients with CKD, ranging from 96 to 7,352 patients per study.

### Evaluation of the Risk of Bias of Selected Studies

**FIGURE 2:**
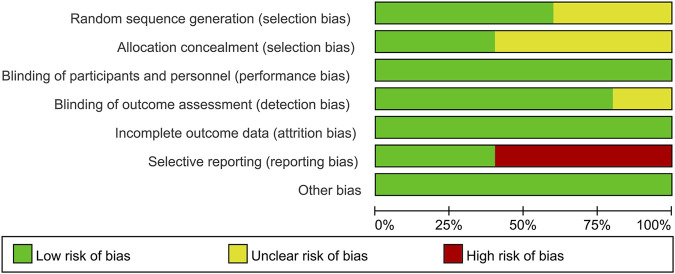
Risk of bias graph: Data for each risk-of-bias item presented as percentages across all included studies.

The risk of bias for the included RCTs was assessed using the Cochrane Risk-of-Bias tool. None of the RCTs had an overall low risk of bias. Two RCTs (Bakris et al., 2020; Pitt et al., 2021) had an unclear risk of bias for sequence generation, three (Pitt et al., 2013; Bakris et al., 2020; Pitt et al., 2021) for allocation concealment, and one (Katayama et al., 2017) for the blinding of outcome assessment. Three RCTs (Bakris et al., 2015; Bakris et al., 2020; Pitt et al., 2021) had high risk of bias for selective reporting, since some outcomes mentioned in a prespecified analysis plan were not reported. All RCTs (Pitt et al., 2013; Bakris et al., 2015; Katayama et al., 2017; Bakris et al., 2020; Pitt et al., 2021) were categorized as low risk of bias for blinding of participants and personnel as well as for incomplete outcome data and other items. The risk of bias assessments of included trials is shown in Figure 2.

### Meta-Analysis

#### Urinary Albumin-to-Creatinine Ratio Mean Ratio From Baseline

**FIGURE 3:**
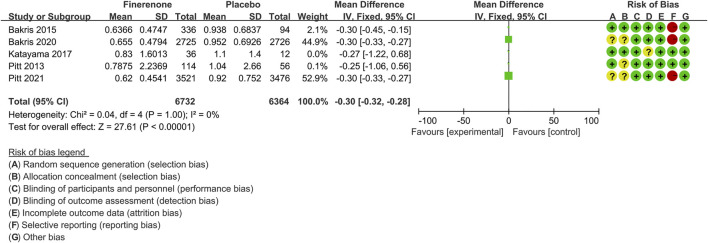
Meta-analysis results of mean of the UACR from the baseline for finerenone and placebo patient groups.

Five trials (Pitt et al., 2013; Bakris et al., 2015; Katayama et al., 2017; Bakris et al., 2020; Pitt et al., 2021) compared the urinary albumin-to-creatinine ratio (UACR) of finerenone (n = 6,732) versus placebo (n = 6,364) groups of CKD patients. As shown in Figure 3, the UACR mean from baseline was significantly lower for finerenone than for placebo [MD −0.30, 95% CI (−0.32, −0.28), *p* < 0.00001].

#### Changes in Estimated Glomerular Filtration Rate From Baseline

**FIGURE 4:**
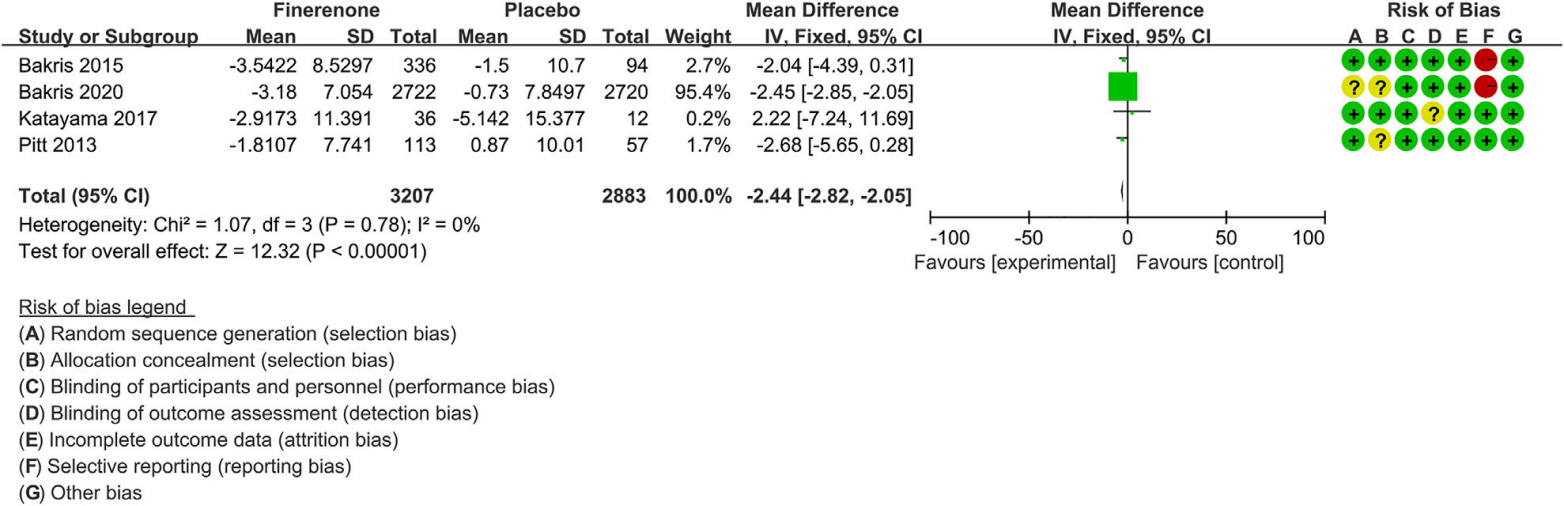
Meta-analysis results of changes in the eGFR from the baseline for finerenone and placebo patient groups.

Four trials (Pitt et al., 2013; Bakris et al., 2015; Katayama et al., 2017; Bakris et al., 2020) compared estimated glomerular filtration rate (eGFR) values between finerenone (n = 3,207) and placebo (n = 2,883) CKD patient groups. As shown in Figure 4, the decrease in eGFR from baseline was significantly higher with finerenone than with placebo [MD −2.44 (95% CI −2.82, −2.05), *p* < 0.00001].

#### Decrease in Estimated Glomerular Filtration Rate ≥40%

**FIGURE 5:**
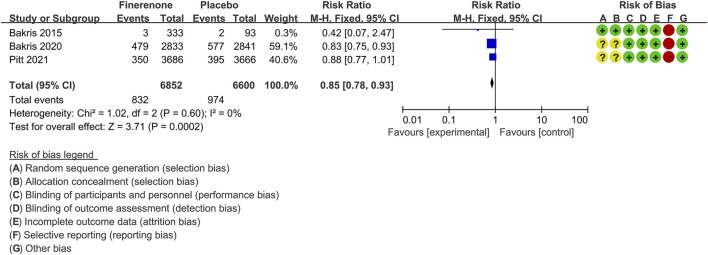
Meta-analysis results of the proportion of patients with ≥40% decrease in the eGFR in finerenone versus placebo patient groups.

Three trials (Bakris et al., 2015; Bakris et al., 2020; Pitt et al., 2021) compared the proportion of patients showing a ≥40% decrease in the eGFR at any time post-baseline between the finerenone (n = 6,852) and placebo (n = 6,600) CKD patient groups. The proportion of patients was significantly lower for the finerenone than for the placebo treatment group [RR 0.85 (95% CI 0.78, 0.93), *p* = 0.0002], as shown in Figure 5.

#### End-Stage Kidney Disease

**FIGURE 6:**
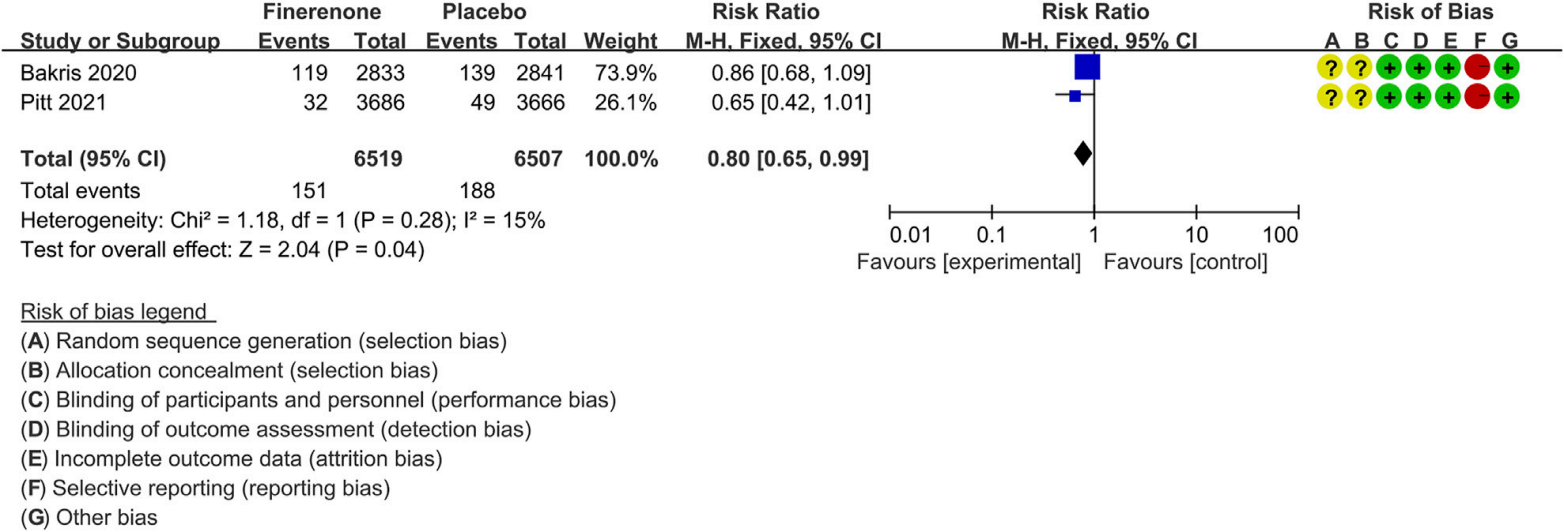
Meta-analysis results of the efficacy of finerenone versus placebo for end-stage kidney disease.

Two trials (Bakris et al., 2020; Pitt et al., 2021) compared the proportion of CKD patients with ESKD following treatment with finerenone (n = 6,519) versus placebo (n = 6,507). The overall results showed a significantly lower proportion of patients with ESKD in the finerenone than in the placebo group [RR 0.80 (95% CI 0.65, 0.99), *p* = 0.04] (Figure 6).

#### Cardiovascular Events

**FIGURE 7:**
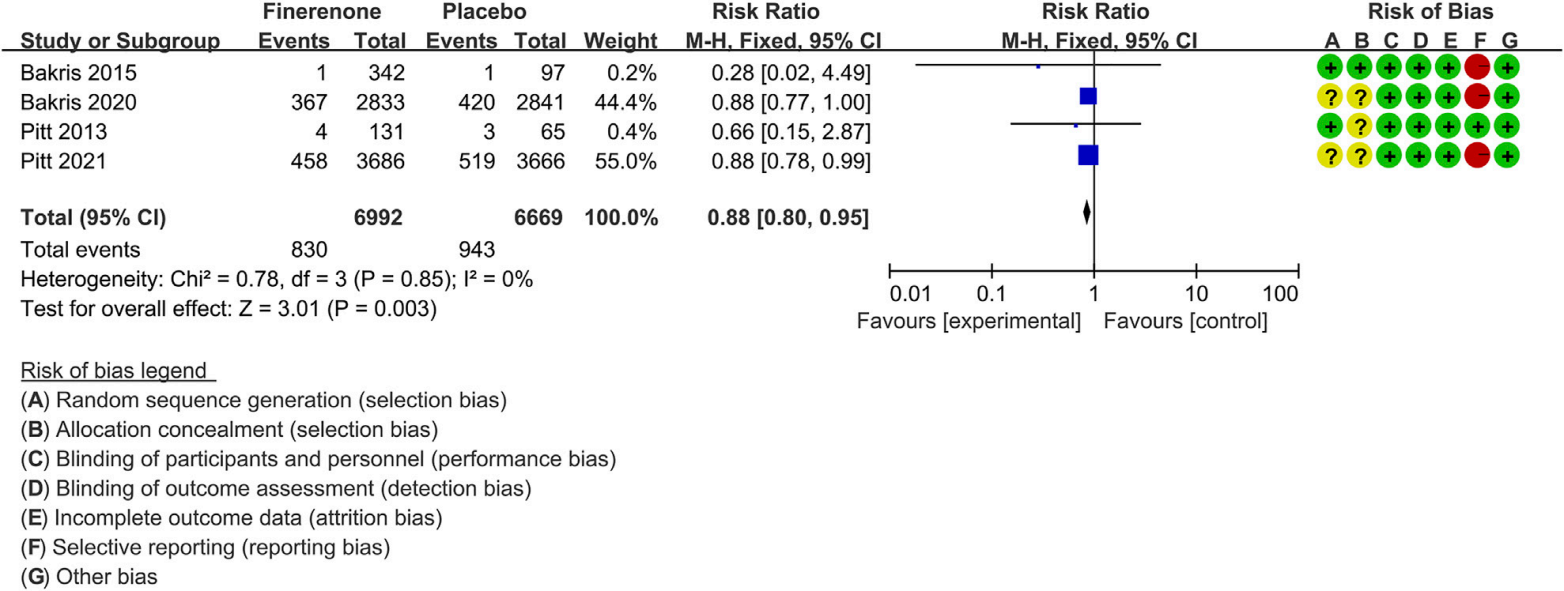
Meta-analysis results of the effect of finerenone versus placebo on cardiovascular events.

Four trials (Pitt et al., 2013; Bakris et al., 2015; Bakris et al., 2020; Pitt et al., 2021) compared the proportion of CKD patients with CVs between groups treated with finerenone (n = 6,992) versus placebo (n = 6,669). As shown in Figure 7, the proportion of patients experiencing CVs was significantly lower in the finerenone than in the placebo group [RR 0.88 (95% CI 0.80, 0.95), *p* = 0.003].

#### Serum Potassium Concentrations

**FIGURE 8:**
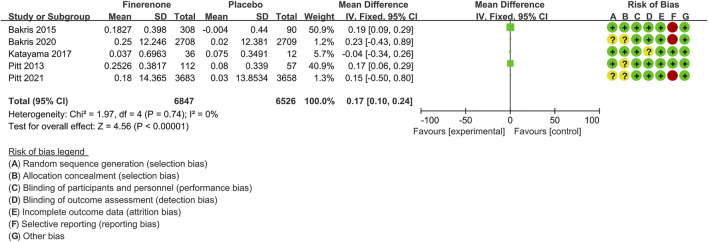
Meta-analysis results of changes in serum potassium concentration between finerenone and placebo patient groups.

Five trials (Pitt et al., 2013; Bakris et al., 2015; Katayama et al., 2017; Bakris et al., 2020; Pitt et al., 2021) compared changes in the serum potassium levels in finerenone-treated (n = 6,847) versus placebo (n = 6,526) CKD patients. As shown in Figure 8, an increase in serum potassium was significantly higher in the finerenone group relative to placebo [MD 0.17 (95% CI 0.10, 0.24), *p* < 0.00001].

#### Hyperkalemia

**FIGURE 9:**
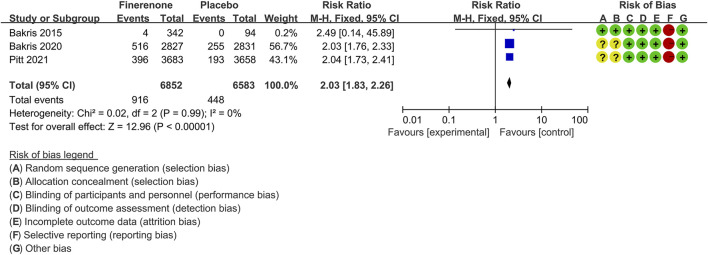
Meta-analysis results of hyperkalemia in finerenone compared to placebo patient groups.

Five trials (Pitt et al., 2013; Bakris et al., 2015; Katayama et al., 2017; Bakris et al., 2020; Pitt et al., 2021) compared the proportion of CKD patients with hyperkalemia in finerenone (n = 6,852) versus placebo (n = 6,583) groups. As shown in Figure 9, the proportion of patients with hyperkalemia was significantly higher in the finerenone than in placebo groups [RR 2.03 (95% CI 1.83, 2.26), *p* < 0.00001].

#### Adverse Events

**FIGURE 10:**
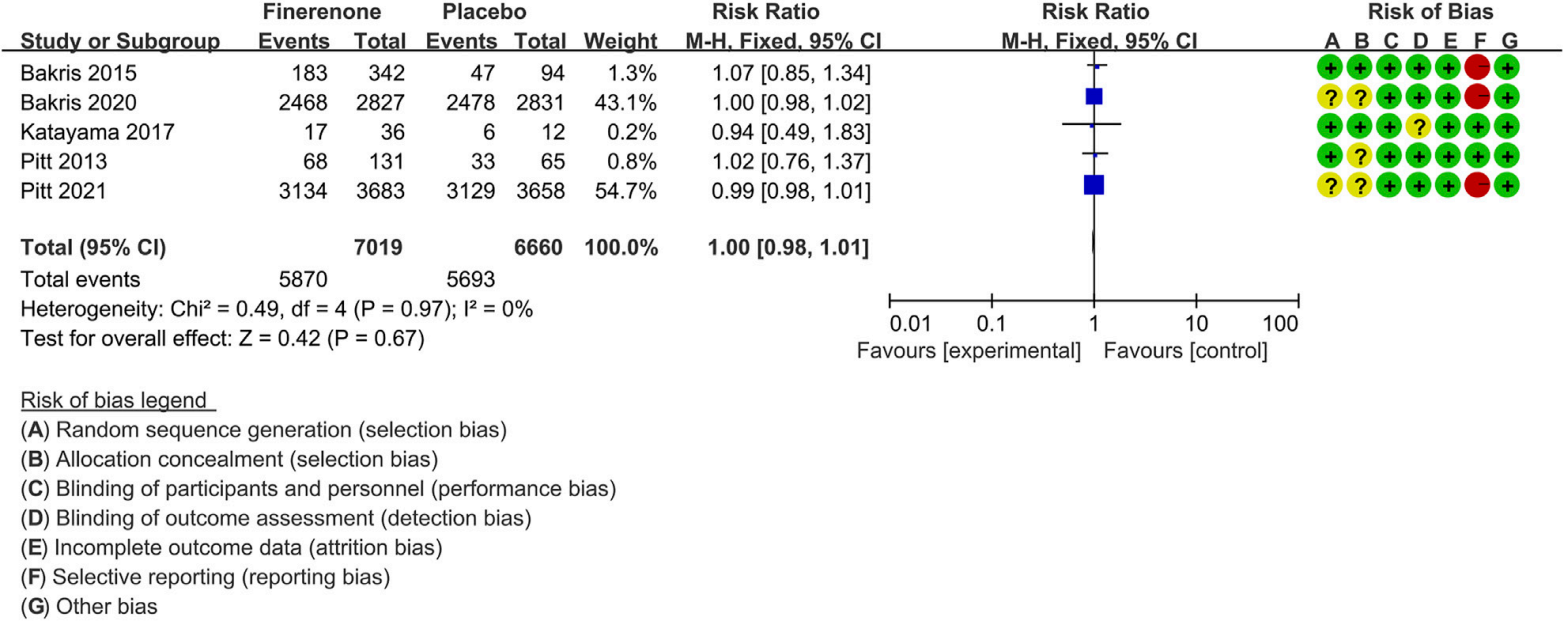
Meta-analysis results of adverse events in finerenone compared to placebo patient groups.

Five trials (Pitt et al., 2013; Bakris et al., 2015; Katayama et al., 2017; Bakris et al., 2020; Pitt et al., 2021) compared the proportion of CKD patients with AEs in finerenone (n = 7,019) versus placebo (n = 6,660) groups. As shown in Figure 10, the proportion did not show any difference in finerenone and placebo [RR 1.00 (95% CI 0.98–1.01), *p* = 0.67].

### Certainty of Evidence

All outcome indicators were evaluated using GRADEproGDT. The quality of evidence was downgraded for the risk of bias and upgraded for the large effect. After comprehensive analysis, the summary of findings table was formed, and it was found that all outcome indicators were of moderate quality or high quality (Supplementary Table S4).

### Publication Bias

Publication bias was not examined because all outcome indicators were observed for less than 10 studies.

## Discussion

The current meta-analysis included studies that used doses of at least 10 mg/day finerenone to assess its overall efficacy and safety, with a view to establishing its optimal potential effects (Snelder et al., 2020). The large sample size of the current meta-analysis facilitated the assimilation of evidence obtained from ongoing clinical trials on the efficacy and safety of finerenone for patients with CKD. The overall results showed that for CKD patients, the UACR mean from baseline was decreased significantly in the finerenone relative to the placebo group. The proportion of patients with a ≥40% decrease in the eGFR at any time post-baseline was markedly lower, though changes in the eGFR from the baseline were reduced significantly in the finerenone relative to the placebo group. Moreover, the proportion of patients with ESKD and CVs was significantly lower for finerenone than that for placebo treatment groups. However, an increase in the serum potassium concentration and the incidence of hyperkalemia were significantly higher in the finerenone group. The incidence of AEs was similar between the groups. In all cases, results were rated as providing moderate-quality or high-quality evidence.

Published studies indicate a robust relationship between albuminuria reduction and slowing of CKD progression as well as reduced cardiovascular and heart failure risk (Schmieder et al., 2014; Yamout et al., 2014; Bakris et al., 2015). A study by Snelder (Snelder et al., 2020) showed that the UACR decreases with increasing finerenone concentrations. Doses of 10–20 mg once daily appeared safe and efficacious in reducing albuminuria. Data from laboratory models indicate that decreased UACR is the result of complex anti-inflammatory and antifibrotic effects through the inhibition of overactivation of the MR (Lattenist et al., 2017; Barrera-Chimal et al., 2018; Grune et al., 2018; Agarwal et al., 2021c). Finerenone is structurally distinct from steroidal MRAs in view of the lack of a steroidal nucleus and cofactor that binds to yield a relatively more pronounced antifibrotic effect (Kolkhof et al., 2014; Grune et al., 2018). These studies’ results are consistent with the findings of our meta-analysis. It should be noted that screening for albuminuria to identify at-risk patients among patients with type 2 diabetes facilitates reduction of both cardiovascular and kidney disease burden (Agarwal et al., 2021a).

Although our meta-analysis found that changes in the eGFR from the baseline were reduced significantly in the finerenone relative to the placebo group. Bakris’s study (Bakris et al., 2020) showed the that eGFR decreased more slowly with finerenone than with placebo after 4 months; the decrease in eGFR was smaller with finerenone after about 26 months. In addition to a regulatory role in electrolytes and fluid homeostasis in the kidney, MR activation exerts direct effects on the heart and vasculature, including stimulation of inflammation, collagen formation, fibrosis, and necrosis (Lattenist et al., 2017; Epstein, 2021). Finerenone controls inflammation by promoting the expression of M2-anti-inflamatory markers and reducing the population of inflammatory CD11b+, F4/80+, and Ly6C^high^ macrophages (Barrera-Chimal et al., 2018). Finerenone has additionally been shown to improve endothelial dysfunction through enhancing nitric oxide (NO) bioavailability and decreasing superoxide anion levels based on the upregulation of vascular and renal superoxide dismutase activity in rats with chronic kidney disease (González-Blázquez et al., 2018). The direct antifibrotic properties of finerenone resulted in reduced myofibroblast and collagen deposition accompanied by a decrease in kidney plasminogen activator inhibitor (PAI)-1 and naked cuticle 2 (NKD2) expression in mouse models of progressive kidney fibrosis (Droebner et al., 2021). Our data showing that the relative risk of a sustained ≥40% decrease in the eGFR was reduced by 15%, ESKD by 20%, and FIDELITY analysis showed the relative risk of a sustained ≥57% decrease in the eGFR was reduced by 30% (Agarwal et al., 2021a). These results indicate that finerenone may have long-term renal benefits for CKD patients. Reduction of these outcomes, which is of great relevance to both patients and payers, is therefore notable.

The FIDELIO-DKD trial found that finerenone had a beneficial effect on the overall risk of CVs, regardless of preexisting CVD status among patients with chronic kidney disease and T2DM (Filippatos et al., 2021). Distinct from steroidal MRAs, macrophage invasion is reported to be potently blocked by finerenone while eplerenones exert no significant effect. In another earlier study, finerenone potently inhibited aldosterone-induced profibrotic gene tenascin-X (TNX) expression in a rat cardiomyocytic cell line whereas eplerenone exhibited only weak activity and spironolactone treatment did not result in the significant inhibition (Grune et al., 2018). Finerenone treatment induced a distinct cardiac gene expression profile, including differential expression of brain natriuretic peptide (BNP) and troponin T type 2 (Tnnt2), as well as significant reduction of transverse aortic constriction (TAC)-induced left ventricular (LV) wall thickening (assessed via echocardiography), suggesting that its beneficial effects on left ventricular mass development in the pressure overload are associated with the cardiac gene expression profile (Grune et al., 2016). Furthermore, finerenone has been shown to exert favorable vascular effects through restoring vascular integrity and preventing adverse vascular remodeling in different murine models of vascular injury (Dutzmann et al., 2017).

The serum potassium concentrations and incidence of hyperkalemia in the finerenone treatment group were increased relative to those in the placebo group. However, patients with high serum potassium baseline levels (especially >4.8 mmol) and poor renal function had a high risk for hyperkalemia whether in the finerenone groups or placebo groups (Agarwal et al., 2021b; Goulooze et al., 2021). Although finerenone was associated with a higher overall risk of hyperkalemia than placebo, a markedly lower incidence of hyperkalemia and lower mean change of potassium concentrations were observed in the finerenone than in the spironolactone group (Pitt et al., 2013; Kintscher et al., 2021). Since our data showed that finerenone increased kalemia by 0.17 mmol/L, finerenone should not be afraid of the risk of hyperkalemia because of the potential benefits of taking it (Baran et al., 2021). However, Randomized Aldactone Evaluation Study (RALES) cautions us about what also might come following the publication of impactful results from controlled trials (Moura-Neto and Ronco, 2021). Therefore, routine potassium monitoring and hyperkalemia management strategies [finerenone treatment interruption and dose reduction or adding other therapies (such as diuretics or potassium binders)] are essential, which will minimize the impact of hyperkalemia and enable sustained clinical use of finerenone (Clase et al., 2020; Agarwal et al., 2021a; Epstein et al., 2021; Moura-Neto and Ronco, 2021). Notably, AEs are not all related to the trial regimen, although our meta-analysis showed a comparable incidence of AEs between the finerenone and placebo groups.

Our study has a number of limitations that should be considered. First, we employed only published reports and could not entirely avoid potential publishing bias for negative results and small-scale studies. Second, this meta-analysis was limited by the relatively small number of studies (we included only three phase 2 and two phase 3 studies), and three of these five included trials are extremely small, only accounting for about 5% (682/13,078) of the total samples size. Third, the control intervention in our meta-analysis was only placebo, and no data were extracted from studies with positive drug controls. Further research is warranted to provide definitive evidence regarding the efficacy and safety of finerenone in comparison to the positive drug including spironolactone and eplerenone (ongoing RCTs about finerenone: NCT05047263, NCT05013008, and NCT04477707).

## Conclusions

Data from our meta-analysis suggest that finerenone confers significant antiproteinuric benefits in patients with CKD, with a favorable effect on a decrease in the eGFR (≥40%). The use of finerenone resulted in lower incidence of ESRD and CVs in patients with CKD. Although a higher risk of hyperkalemia was observed with finerenone than with placebo, discontinuation of trial regimens owing to hyperkalemia was infrequent. No significant differences in AEs between finerenone and placebo groups were evident. The observed benefits of finerenone were clinically significant and did not cause unacceptable side effects. Our collective findings support the utility of finerenone as a novel promising therapeutic tool for patients with CKD.

## Data Availability

The original contributions presented in the study are included in the article/Supplementary Material; further inquiries can be directed to the corresponding author.
