# Supplementary material for: Efficacy and Safety of Finerenone in Chronic Kidney Disease: A Systematic Review and Meta-Analysis of Randomized Clinical Trials
Source: Front Pharmacol. 2022 Feb 7;13:819327. doi: 10.3389/fphar.2022.819327 (PMC8859447; doi:10.3389/fphar.2022.819327)
Supplement: Supplementary file 2 [file Table2.docx]

| **Our search strategy is as follows, taking it in Pubmed for example:** |
| --- |
| (finerenone* OR kerendia* OR BAY 94-8862*) AND (Renal Insufficiency, Chronic[mh] OR Chronic Renal Insufficiencies* OR Renal Insufficiencies, Chronic* OR Chronic Renal Insufficiency* OR Kidney Insufficiency, Chronic* OR Chronic Kidney Insufficiency* OR Chronic Kidney Insufficiencies* OR Kidney Insufficiencies, Chronic* OR Chronic Kidney Diseases* OR Chronic Kidney Disease* OR Disease, Chronic Kidney* OR Diseases, Chronic Kidney* OR Kidney Disease, Chronic* OR Kidney Diseases, Chronic* OR Chronic Renal Diseases* OR Chronic Renal Disease* OR Disease, Chronic Renal* OR Diseases, Chronic Renal* OR Renal Disease, Chronic* OR Renal Diseases, Chronic*) AND (randomized controlled trial[pt] OR controlled clinical trial[pt] OR randomized[tiab] OR placebo[tiab] OR drug therapy[sh] OR randomly[tiab] OR trial[tiab] OR groups[tiab] NOT (animals [mh] NOT humans [mh])) |
