# Supplementary material for: Efficacy and Safety of Finerenone in Chronic Kidney Disease: A Systematic Review and Meta-Analysis of Randomized Clinical Trials
Source: Front Pharmacol. 2022 Feb 7;13:819327. doi: 10.3389/fphar.2022.819327 (PMC8859447; doi:10.3389/fphar.2022.819327)
Supplement: Supplementary file 3 [file Table3.docx]

| Study ID | Location | Disease | Age(years) | No.of patients(M/F) | Intervention  (Dosage regimen) | Period  (Intervention+follow-up) | clinical trial | baseline eGFR  (Mean (SD))  (ml/min/1.73m^2^) | Baseline UACR (mg/g) |
| --- | --- | --- | --- | --- | --- | --- | --- | --- | --- |
| Pitt  2013 | 55 centers in 10 countries | HFrEF (heart failure with reduced ejection fraction)  and mild/moderate CKD | Mean (range)  Part A:  66.30 (42-85)  Part B:  72.10 (40–89) | Part A:  65(52/13)  Part B:  392(312/80) | Part A:  Finerenone  (2.5mg,5mg,10mg qd) and placebo  Part B: finerenone  (2.5mg,5mg, 10mg, qd, 5mg bid)  open-label spironolactone (25mg,50mg, qd) and placebo | 28+14 days | phase II | PartA:  69.10 (8.43)  PartB:  47.00 (10.00) | Geometric mean (geometric SD)  Part A：  13.67 (3.2)  Part B：  21.33 (4.87) |
| Bakris  2015 | 148 sites in 23 countries | Diabetic Nephrop-athy(DN) | Mean (SD)  T:64.32 (9.21)  C:63.26 (8.68) | T:727 (570/127)  C:94 (69/25) | T: finerenone  (1.25mg, 2.5mg, 5mg, 7.5mg, 10mg, 15mg, 20mg qd)  C: placebo | 90+30 days | phase 2B | T:66.94 (21.88)  C:77.21 (20.43) | Geometric mean  (range)  T：201.0 (4.4-4948)  C: 188.4 (15.0-3056) |
| Katayama 2017 | 16 centers in Japan | T2DM  and DN | Mean (SD)  T:62.40 (9.79)  C:66.75 (9.02) | T: 84 (67/17)  C: 12 (10/2) | T: finerenone  (1.25mg,2.5mg, 5mg, 7.5mg, 10mg, 15mg, 20mg, qd)  C: placebo | 90+30 days | phase 2B | T:65.23 (13.70)  C:60.88 (16.53) | Geometric mean (range)  T: 217.66  (9.4-2404.4)  C: 256.80  (28.9–1338.1) |
| Bakris  2020 | 978 study location in 48 countries | CKD and T2DM | Mean (SD)  T:65.4 (8.9)  C:65.7 (9.2) | T: 2833 (1953/880)  C: 2841 (2030/811) | T: Finerenone  (10mg,20mg qd)  C: placebo | 32 months+4 weeks  and 5 days | Phase 3 | T:44.4 (12.5)  C:44.3 (12.6) | Median [interquartile rang (IQR)]:  T: 833  (441–1628)  C: 867  (453–1645) |
| Pitt 2021 | 976 study locations  in 48 countries | CKD andT2DM | Mean (SD)  T:64.1 (9.7)  C:64.1 (10.0) | T: 3686(2528/1158)  C: 3666(2577/1089) | T: Finerenone  (10mg,20mg qd)  C: placebo | 41 months+4 weeks  and 5 days | Phase 3 | T:67.6 (21.7)  C:68.0 (21.7) | Median (IQR)  T: 302  (105–749)  C: 315  (111–731) |
