# Supplementary material for: Efficacy and Safety of Finerenone in Chronic Kidney Disease: A Systematic Review and Meta-Analysis of Randomized Clinical Trials
Source: Front Pharmacol. 2022 Feb 7;13:819327. doi: 10.3389/fphar.2022.819327 (PMC8859447; doi:10.3389/fphar.2022.819327)
Supplement: Supplementary file 4 [file Table4.docx]

| **Finerenone compared to placebo for Chronic Kidney Disease** | | | | | |
| --- | --- | --- | --- | --- | --- |
| Outcomes | **Anticipated absolute effects^*^** (95% CI) | | Relative effect (95% CI) | № of participants (studies) | Certainty of the evidence (GRADE) |
|  | **Risk with placebo** | **Risk with Finerenone** |  |  |  |
| decrease in eGFR | / | MD **2.44 lower** (2.82 lower to 2.05 lower) | - | 6090 (4 RCTs) | ⨁⨁⨁◯ Moderate^a^ |
| mean UACR ratio to baseline | / | MD **0.3 lower** (0.32 lower to 0.28 lower) | - | 13096 (5 RCTs) | ⨁⨁⨁◯ Moderate^a^ |
| decrease of ≥40% in eGFRfrom baseline | 148 per 1,000 | **125 per 1,000** (115 to 137) | **RR 0.85** (0.78 to 0.93) | 13452 (3 RCTs) | ⨁⨁⨁◯ Moderate^a^ |
| cardiovascular events | 141 per 1,000 | **124 per 1,000** (113 to 134) | **RR 0.88** (0.80 to 0.95) | 13661 (4 RCTs) | ⨁⨁⨁◯ Moderate^a^ |
| End-stage kidney disease | 29 per 1,000 | **23 per 1,000** (19 to 29) | **RR 0.80** (0.65 to 0.99) | 13026 (2 RCTs) | ⨁⨁⨁◯ Moderate^a^ |
| change in serum potassium | / | MD **0.17 higher** (0.1 higher to 0.24 higher) | - | 13373 (5 RCTs) | ⨁⨁⨁◯ Moderate^a^ |
| hyperkalemia | 68 per 1,000 | **138 per 1,000** (125 to 154) | **RR 2.03** (1.83 to 2.26) | 13435 (3 RCTs) | ⨁⨁⨁⨁ High^a, b^ |
| adverse events | 855 per 1,000 | **855 per 1,000** (838 to 863) | **RR 1.00** (0.98 to 1.01) | 13679 (5 RCTs) | ⨁⨁⨁◯ Moderate^a^ |
| ***The risk in the intervention group** (and its 95% confidence interval) is based on the assumed risk in the comparison group and the **relative effect** of the intervention (and its 95% CI).  **CI:** confidence interval; **MD:** mean difference; **RR:** risk ratio | | | | | |

a. Risk of bias

b. Large effect
